# Regulatory T cells in primary sjögren disease: regulatory mechanisms and therapeutic prospects

**DOI:** 10.3389/fimmu.2026.1802660

**Published:** 2026-04-15

**Authors:** Wang Chengzhi, Liu Yifan, Li Songwei, Du Mengmeng, Zhu Keying, Li Huan

**Affiliations:** 1 Department of Rheumatology, The First Affiliated Hospital of Henan University of Chinese Medicine, Zhengzhou, China; 2 The First Clinical College of Henan University of Chinese Medicine, Zhengzhou, China; 3 The Second Affiliated Hospital of Henan University of Chinese Medicine, Zhengzhou, China

**Keywords:** pathogenic mechanism, primary sjögren disease, regulatory T cells, Th17/Treg immune balance, treatment prospects

## Abstract

Primary Sjögren disease (SjD) is a chronic inflammatory autoimmune disorder characterized by lymphocyte proliferation and progressive damage to exocrine glands. Its pathogenesis is complex, and clinical treatment remains challenging. Regulatory T cells (Tregs), a subset of inhibitory T lymphocytes, play a pivotal role in maintaining peripheral immune tolerance and immune homeostasis. They are also critically involved in the pathogenesis and progression of various autoimmune diseases, including SjD. Consequently, modulating the proliferation, activation, and functional balance of Tregs holds significant promise for ameliorating the immune-inflammatory microenvironment in SjD and slowing disease progression. Recent studies have shown that mesenchymal stem cells (MSCs), fenofibrate, and metformin can promote Treg proliferation and improve their function, thereby restoring the Th17/Treg immune balance. These interventions synergistically reduce inflammatory responses, downregulate abnormal immune activation, and alleviate tissue pathological damage, ultimately leading to significantly increased saliva and tear secretion. This review summarizes the regulatory mechanisms of Tregs in SjD based on recent literature and explores the potential of Treg-targeted therapeutic strategies for SjD, aiming to provide a theoretical basis for the development of novel treatment approaches for this disease.

## 1 Introductions

Primary Sjögren disease (SjD) is a chronic autoimmune disorder primarily characterized by dry eyes and dry mouth. It may also present with systemic manifestations such as fatigue, elevated immunoglobulin levels, and positivity for various autoantibodies. In severe cases, the disease can affect multiple organ systems and significantly impair patients’ quality of life (1). Although the pathogenesis of SjD remains incompletely understood, it is widely recognized to be strongly associated with immune dysregulation and inflammatory responses (2). Among these mechanisms, the T-cell-mediated inflammatory response and immune imbalance serve as central drivers in the onset and progression of SjD. Multiple T-cell subsets contribute to tissue damage and glandular secretory dysfunction through the activation and inhibition of regulatory T cells (Tregs) (3). In clinical practice, SjD management primarily relies on immunosuppressants, glucocorticoids, and biologics. With ongoing research advances, several novel immunomodulatory therapies have emerged in recent years, including cytokine-based treatments, gene chip technology, gene editing, and stem cell transplantation. However, these emerging approaches continue to face widespread challenges such as high cost, frequent adverse effects, and poor patient adherence (4). In recent years, the abnormal quantity and function of Tregs in SjD have received increasing attention. Therapeutic strategies targeting Tregs have shown unique prospects. Among them, the mammalian target of rapamycin (mTOR) inhibitor sirolimus (Rapamycin) has emerged as a promising direction for in-depth exploration in the immunomodulatory treatment of SjD. This is because it exerts multiple effects, including enhancing Treg function, inhibiting abnormal lymphocyte proliferation, and inducing apoptosis, while also being effective, well-tolerated, and amenable to dose adjustment. Notably, sirolimus can reduce the expansion of double-negative T cells (DNTs), thereby correcting the immune imbalance present in SjD from multiple aspects (5, 6). Therefore, with a deeper understanding of the immunological mechanisms underlying SjD, developing new therapeutic strategies that are precisely targeted, safe, and cost-effective has become a crucial scientific challenge in the field. A thorough elucidation of the role of Tregs in the pathogenesis and progression of the disease may provide an important theoretical foundation for refining future treatment approaches.

## 2 Overview of Tregs

### 2.1 Differentiation and development of Tregs

Regulatory T cells (Tregs) are a functionally heterogeneous subset of T lymphocytes with potent immunosuppressive activity. They are critical for establishing and maintaining peripheral immune tolerance and overall immune homeostasis. The differentiation and development of Tregs are tightly regulated and occur primarily via two distinct pathways: thymus-derived differentiation and peripheral induction (7). Thymus-derived regulatory T cells (tTregs) predominantly undergo development and maturation in the thymus. Their differentiation is initiated by a moderate-affinity interaction between the T cell receptor of CD4+ single-positive T cells and self-peptide-MHC class II complexes presented on thymic stromal cells. The key regulator of this process is the transcription factor Foxp3, whose expression not only directs Tregs functional specialization but also critically determines their phenotypic stability and long-term functional maintenance (8, 9). In contrast, peripheral regulatory T cells (pTregs) arise from naïve CD4+ T cells in peripheral tissues under specific microenvironmental conditions. Their differentiation relies on the coordinated integration of three critical signals: (1) Cytokine signaling, particularly high concentrations of TGF-β combined with IL-2, which drives the expression of the lineage-defining transcription factor Foxp3; (2) Persistent, low-level antigenic stimulation; and (3) Co-stimulatory signals delivered by tolerogenic antigen-presenting cells (such as dendritic cells expressing ICOS-L, PD-L1, and other inhibitory ligands) (10, 11). Based on their functional status, Tregs can be classified into resting Tregs and activated/memory Tregs. The former exhibit a basal inhibitory state, whereas the latter acquire an enhanced functional capacity following strong antigen stimulation. In an inflammatory environment, resting Tregs can differentiate into activated Tregs and subsequently migrate to the inflammatory site to exert local immune regulatory effects (12, 13).

**Figure 1:**
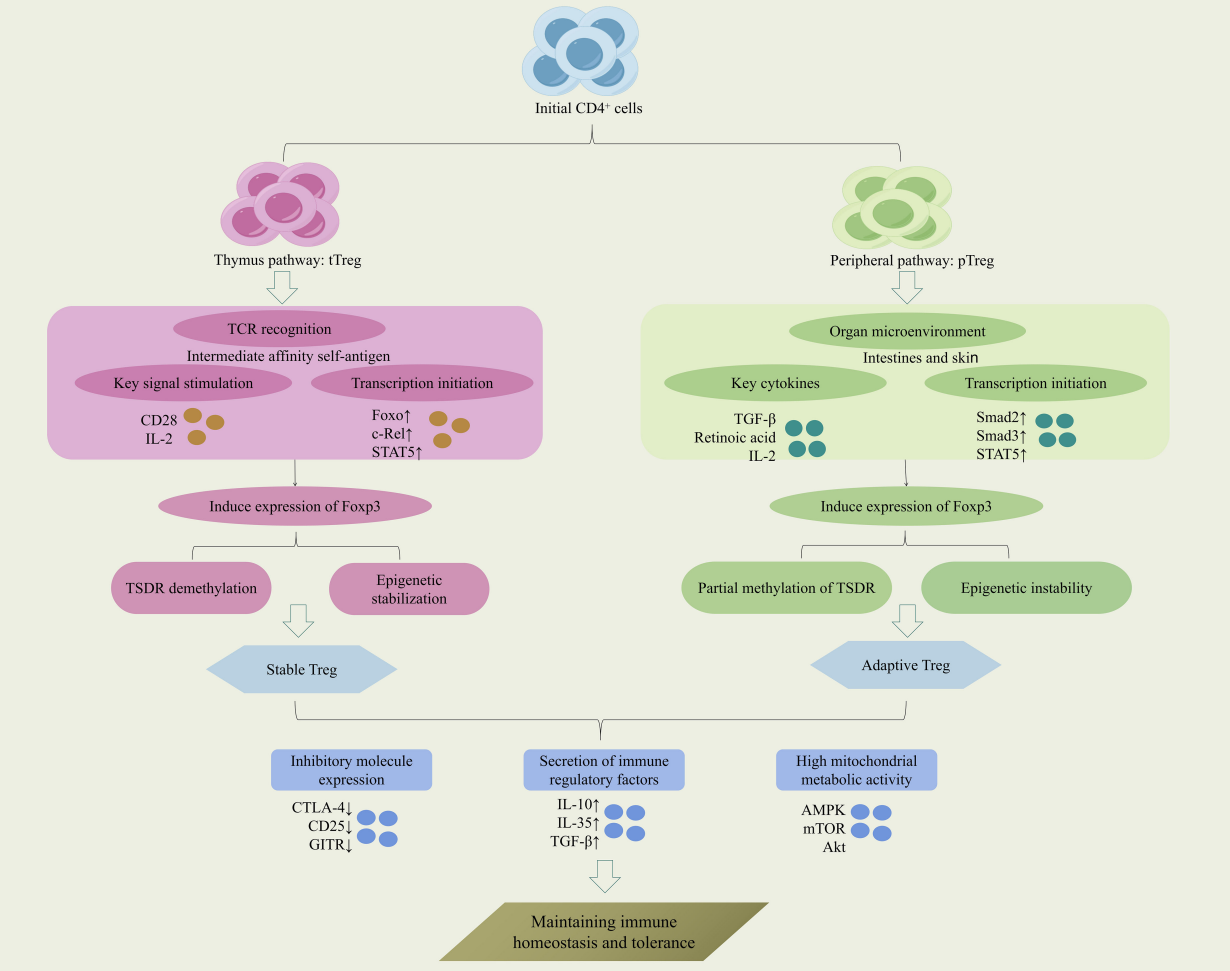
Mechanisms of Treg differentiation and development.

However, the dynamic ratio of activated to quiescent Tregs in physiological and pathological conditions remains controversial. Some studies report a significant increase in activated Treg proportions in the peripheral blood of patients with autoimmune diseases, suggesting a compensatory mechanism to curb excessive inflammation. Conversely, other evidence shows that activated Tregs are enriched in the tumor microenvironment, where they correlate with enhanced immunosuppression and poor prognosis. This discrepancy may stem from differences in tissue microenvironments and inconsistent selection of phenotypic markers, complicating direct cross-study comparisons. Thus, focusing solely on proportional imbalances between these two subsets is insufficient to elucidate their functional status. Future studies should integrate spatial localization with functional lineage analysis to accurately interpret their immunomodulatory roles.

(The differentiation and developmental mechanisms of Tregs are illustrated in **Figure 1**).

### 2.2 Physiology and pathology of Tregs

Under physiological conditions, Tregs act as central regulators in maintaining immune tolerance and systemic immune homeostasis. They employ diverse mechanisms to exert broad immunosuppressive effects, including the modulation of both physiological and pathological immune responses, the enforcement of self-tolerance to prevent autoimmune dysregulation, and the active support of tissue integrity and barrier function (14). Their mechanisms of action involve not only the secretion of inhibitory cytokines such as IL-10 and TGF-β but also rely on the expression of multiple co-inhibitory receptors on their surface. Through these molecules, Tregs directly regulate intercellular immune interactions, thereby sustaining the dynamic balance of the immune environment (15). Specifically, Tregs effectively inhibit the overactivation and aberrant function of effector T cells and B cells through the expression of inhibitory surface molecules, thereby preventing uncontrolled immune responses and limiting the spread of inflammation (16). Under pathological conditions, impaired immunosuppression may result from declined Treg function, reduced cell numbers, or unstable expression of the transcription factor Foxp3. Such regulatory failure disrupts the balance between effector T cells and B cells, thereby triggering or exacerbating autoimmune diseases (17). The balance between T helper 17 (Th17) cells and Tregs is crucial for maintaining the body’s immune homeostasis. Under normal physiological conditions, Th17 cells and Tregs exist in a dynamic equilibrium. Disruption of this balance—due to increased inflammatory mediators, dysregulated cytokine expression, or defective transcription factors—can lead to various immune disorders (18). Restoring the Th17/Treg immune balance by increasing the proportion of Tregs and reducing that of Th17 cells can help reestablish immune tolerance and promote the repair of damaged tissues (19). In summary, under physiological conditions, Tregs constitute a fundamental pillar of immune tolerance and homeostasis, orchestrating the delicate balance between immune defense and self-protection. In pathological contexts, however, impaired Treg function or reduced Treg numbers may contribute to the development of autoimmune diseases. Conversely, excessive Treg activity or their abnormal accumulation in specific tissues can facilitate tumor immune evasion and sustain chronic infections.

### 2.3 Overview of T lymphocyte subsets

T lymphocyte subsets refer to the groups of T cells classified based on their surface markers and functional differences. They mainly consist of two categories: CD4+ helper T cells and CD8+ cytotoxic T cells (20). Among them, CD4+ T cells further differentiate into functional subgroups such as Th1 (involved in cellular immunity), Th2 (involved in humoral immunity and allergies), Th17 (antagonizing extracellular bacteria but excessive activation can lead to autoimmune diseases), and Treg (regulatory T cells, inhibiting immune responses to maintain tolerance). CD8+ T cells mainly exert their effects by killing infected or cancerous cells (21, 22). Clinically, the counts of CD3+, CD4+, and CD8+ cells and the CD4/CD8 ratio are detected using flow cytometry to assess the immune status of the body. A decreased ratio is commonly seen in immune deficiencies (such as AIDS and malignant tumors), while an increased ratio indicates hyperimmune state (such as autoimmune diseases and transplant rejection). The dynamic balance among these subsets determines whether the immune system is in a defensive, tolerant, or pathological state (23). In addition, double-negative T (DNT) cells are a unique subset of T cells that lack CD4 and CD8 expression. They exhibit bidirectional regulatory functions in autoimmune diseases (24). On the one hand, certain subsets of DNT cells can suppress immune responses by secreting inhibitory cytokines such as IL-35 and TGF-β or by directly killing autoreactive T cells; on the other hand, the abnormal expansion of DNT cells may also promote the progression of inflammation (25). Currently, the specific role of DNT cells in diseases such as SjD remains unclear, and the interaction and functional complementarity between DNT cells and Tregs also require further elucidation (26). Therefore, conducting systematic phenotypic analysis and functional characterization of all lymphocyte subsets, including DNT cells, will help construct a comprehensive immune regulatory network and provide more precise strategies for targeted immune intervention.

## 3 The relationship between Tregs and SjD

### 3.1 Mechanisms of Treg-mediated regulation in SjD

**Figure 2:**
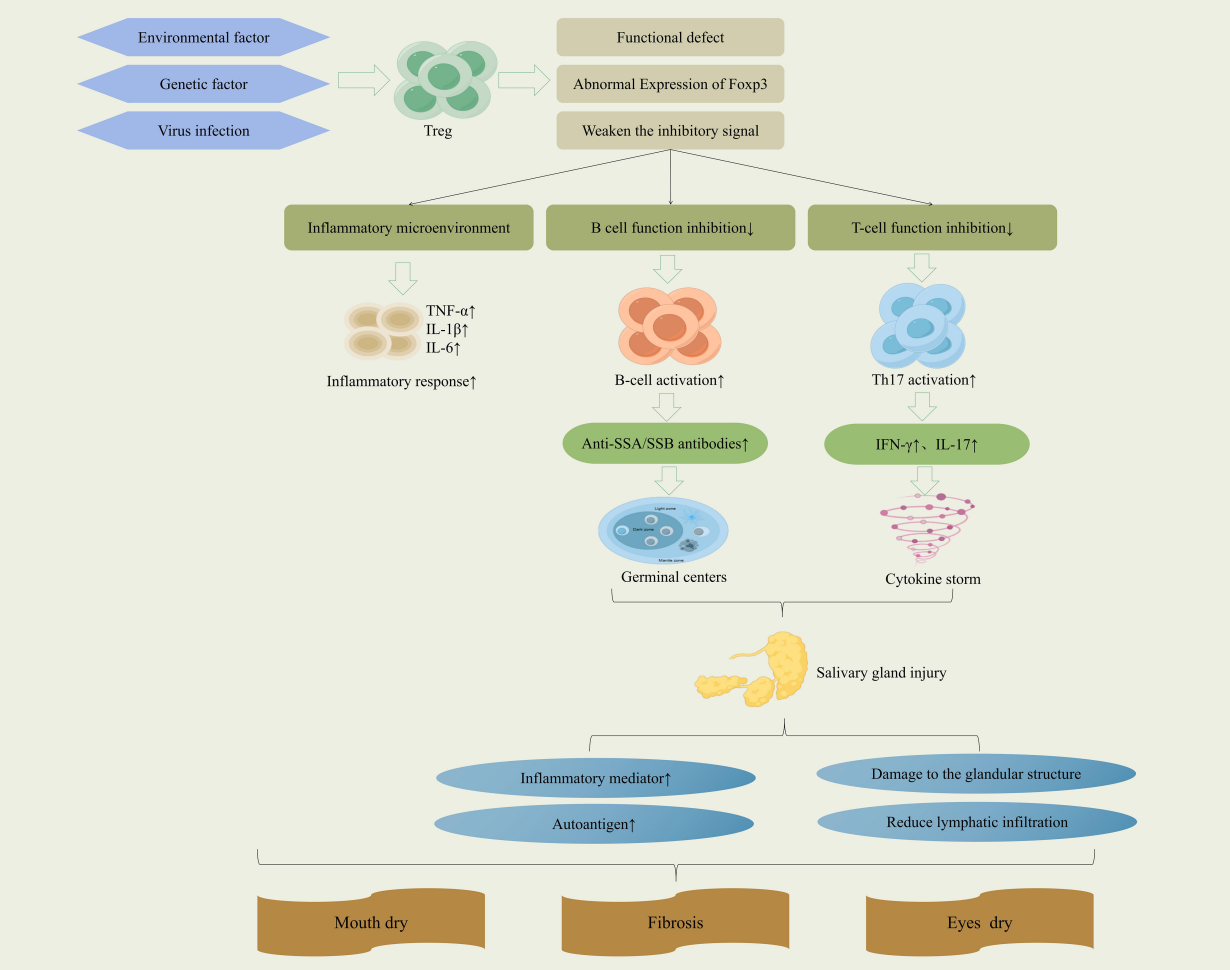
Mechanisms of Treg regulation in SjD.

In SjD pathogenesis, Tregs serve as central regulators of immune homeostasis. A reduction in Treg numbers or impairment in their function weakens the immunosuppressive control over effector T cells and B cells, triggering aberrant immune attacks against exocrine glands. The resulting inflammatory microenvironment within damaged glands is rich in pro-inflammatory cytokines such as IL-1β and TNF-α. These mediators further inhibit Treg differentiation and suppressive function, establishing a self-reinforcing cycle in which diminished Treg activity, heightened immune attack, and a deteriorating inflammatory milieu perpetuate one another. This vicious cycle ultimately drives the chronic progression of SjD and the progressive loss of glandular function (27). The impaired Tregs are unable to effectively inhibit the excessive activation and proliferation of B cells, promoting their differentiation into autoantibody-secreting plasma cells, thereby exacerbating the humoral immune dysregulation. Concurrently, the functional defects of Tregs affect the polarization of innate immune cells such as macrophages, driving their conversion toward pro-inflammatory phenotypes (e.g., M1 type), which continuously release inflammatory factors and amplify tissue inflammation. In SjD, Tregs exhibit functional defects, resulting in a diminished inhibitory effect on pathogenic Th1 and Th17 cells. The consequent persistent elevation of inflammatory cytokines, including IFN-γ and IL-17A, can indirectly upregulate the expression and activity of local matrix metalloproteinase 3 (MMP-3) and MMP-9, thereby contributing to glandular destruction and extracellular matrix remodeling. This process compromises the integrity of glandular structure, promotes the infiltration of inflammatory cells, and directly impairs the function of acini and ducts (14, 28). In summary, Tregs profoundly influence the pathological progression of SjD through multiple mechanisms, including the maintenance of immune tolerance, regulation of innate immunity, and preservation of tissue homeostasis. Their dysfunction serves as a critical link connecting autoimmune attacks to structural damage of exocrine glands. Therefore, targeting the restoration of Tregs function and numbers to disrupt this self-reinforcing pathological cycle represents a promising therapeutic strategy for intervening in the progression of SjD. (The regulatory mechanisms of Tregs in SjD are summarized in **Figure 2**).

### 3.2 The Th17/Treg immune balance in SjD

In SjD, the dynamic balance between Th17 and Treg cells plays a decisive role in disease progression. A hallmark immune characteristic of SjD is the shift of the Th17/Treg immune balance toward a pro-inflammatory state, which disrupts immune homeostasis and sustains chronic inflammation (29). The dynamic regulation between Th17 and Treg cells is essential for maintaining immune tolerance under systemic inflammatory conditions (30). Under normal circumstances, Th17 cells secrete cytokines such as IL-17, IL-22, and IL-23, which drive the recruitment of neutrophils and enhance the local inflammatory response in SjD (31). Tregs mainly exert their extensive immunoregulatory functions by producing inhibitory cytokines such as IL-10 and TGF-β to maintain immune tolerance in SjD (32). In patients with SjD, the Th17/Treg immune balance is significantly disrupted. Specifically, the proportion of Th17 cells in the peripheral blood and target tissues increases, while the number of Tregs decreases significantly (33). Meanwhile, Th17 cells and follicular helper T (Tfh) cells display a state of abnormal activation. The functional imbalance in the Th17/Treg and Tfh/follicular regulatory T (Tfr) cells axes may drive disease progression through several mechanisms, including promoting autoantibody production, sustaining aberrant germinal center activity, and disrupting local immunoregulatory circuits (34). Therefore, targeting the restoration of the Th17/Treg immune balance and reestablishing the physiological homeostasis between immune suppression and inflammatory activation has become the core therapeutic strategy and key intervention direction for improving the immune-inflammatory microenvironment in SjD.

### 3.3 Abnormalities and dysfunction of Tregs in SjD

In SjD, Tregs exhibit reduced numbers, increased apoptosis, and a negative correlation with disease activity. Concurrently, their immunosuppressive function is significantly impaired, as evidenced by decreased secretion of inhibitory cytokines, increased production of pro-inflammatory factors, and an abnormal subset distribution accompanied by an imbalanced Th1/Th17 ratio. Further studies have shown that functional defects of Tregs can directly drive disease onset. Additionally, intestinal dysbiosis may contribute to the disease process by inhibiting Treg development and differentiation. Collectively, these multidimensional disturbances at the levels of quantity, function, and immune regulatory networks constitute the core pathological alterations of Tregs in SjD and represent a critical mechanism underlying immune imbalance in the disease.

#### 3.3.1 Reduced population and impaired survival of Tregs

A study has demonstrated that patients with recurrent SjD exhibit a significant reduction in Treg number in peripheral blood, which is inversely correlated with disease activity. Concurrently, upregulation of the apoptosis executioner protein Caspase-3 is observed in labial gland tissues, indicating enhanced local Treg apoptosis and consequently a marked decrease in Treg population within the tissue as well (35). In patients with SjD, the proportion of Tregs is negatively correlated with CRP, ESR, RF, and IgG levels. Given that these inflammatory markers are typically elevated in individuals with active SjD, this negative correlation suggests that the proportion of Tregs in peripheral blood may be decreased, with the degree of reduction closely associated with disease activity and excessive B cell activation (36). The increase in Tregs in the labial glands of SjD patients is negatively correlated with the number of Tregs in the peripheral blood. This may be related to the anti-inflammatory function of Tregs. As inflammation increases and the cytokine environment becomes conducive to the differentiation and proliferation of Th17 cells, the number of Tregs decreases relatively (37).

#### 3.3.2 Functional and phenotypic deficits in Tregs suppression

In the SjD mouse model, the Tregs in the lymph nodes draining the salivary glands exhibit a state of high activation and low quiescence, with impaired immunosuppressive function: secretion of IL-10 decreases, whereas secretion of pathogenic cytokines such as IFN-γ and IL-17 increases, and the expression of CTLA-4 is upregulated. These Tregs have a weakened ability to inhibit CD4+ T cells *in vitro* and may directly contribute to glandular damage by secreting IFN-γ and IL-17. The study suggests that enhancing the inhibitory function of such Tregs may serve as a potential strategy for treating SjD (38). A study has shown that in the peripheral blood of patients with SjD, the distribution of Tregs and their subgroups is abnormal. Specifically, the ratio of activated Tregs to resting Tregs was negatively correlated with the disease activity score of SjD. Compared with healthy controls, the baseline proportion of resting Tregs in patients is higher and decreases after treatment. Moreover, Tregs may generally exhibit functional inhibition defects (39).

#### 3.3.3 Imbalance of Treg subsets and microenvironment interactions

Mice lacking STIM1/2 specifically in Tregs (*Stim1/2Foxp3 mice*) exhibit SjD-like phenotypes, including salivary gland and lacrimal gland inflammation, secretory dysfunction, and the production of autoantibodies, along with T cell and B cell infiltration and enhanced Th1 immune responses. The adoptive transfer experiment confirmed that this disease is mainly driven by CD4+ T cells that produce IFN-γ: when purified CD4+ T cells from *Stim1/2Foxp3 mice* were transferred to *Rag1^-^/^-^* recipient mice, the recipient mice developed the same disease phenotype, indicating that CD4+ T cells are necessary and sufficient for disease development and are not dependent on B cells or autoantibodies (40, 41). Single-cell RNA sequencing analysis revealed that a similar phenomenon of enhanced Th1 response and weakened memory Treg function is also observed in the peripheral blood mononuclear cells of patients with SjD (42). Furthermore, a study has found that in the peripheral blood and salivary gland tissues of patients with SjD, there is an imbalance in the ratio of Tregs to helper T cell subsets such as Th1, Th2, and Th17, as well as their associated cytokines (43).

#### 3.3.4 Impact of gut microbiota dysbiosis on Tregs

The severity of ocular and systemic symptoms in SjD patients is negatively correlated with the diversity of the intestinal microbiota. The composition of the intestinal microbiota shows an increase in pro-inflammatory bacteria and a decrease in anti-inflammatory bacteria (44). When the intestinal microbiota of SjD patients is transplanted into germ-free mice, it can inhibit the development of CD4+ Foxp3+ Tregs in the ocular draining lymph nodes, thereby damaging ocular surface health (45).

## 4 Targeting Tregs in the treatment of SjD

**Figure 3:**
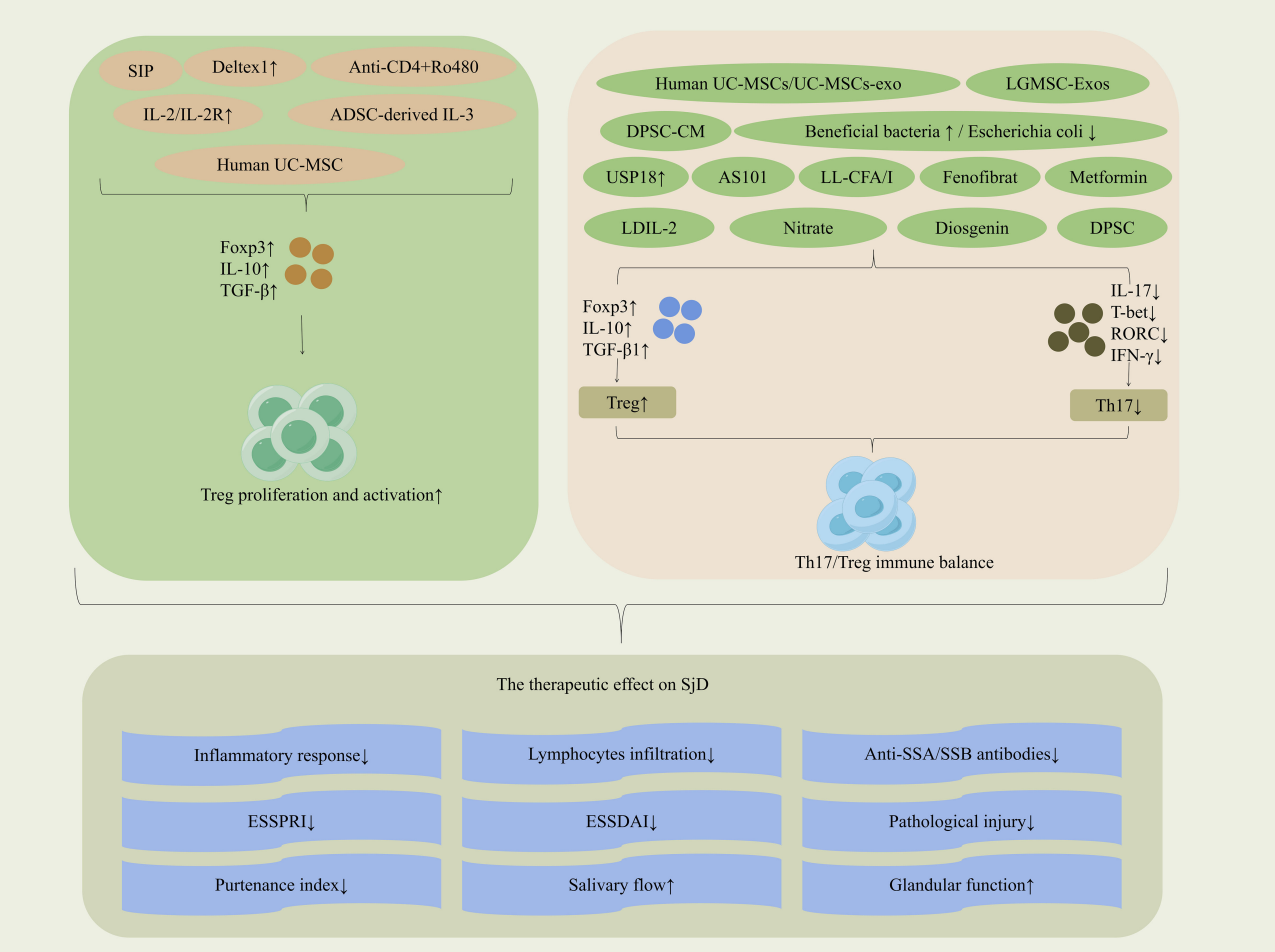
Mechanisms of Treg-targeted therapy in SjD.

The reduction in Treg numbers and their dysfunction serve as initiating and sustained drivers of immune tolerance collapse in SjD. Regulating Treg-related factors can promote their proliferation and activation, restore the Th17/Treg immune balance, and ultimately achieve significant therapeutic effects in SjD. (The mechanisms of regulating Tregs in the treatment of SjD are shown in **Figure 3**).

### 4.1 Regulation of Tregs proliferation and activation in SjD treatment

Sphingolipid metabolism plays a crucial role in the function and differentiation of Tregs (46). The forkhead box protein P3 (Foxp3) can regulate transcription to inhibit the expression of sphingomyelin synthase 1 (Sgms1), leading to the accumulation of ceramides in Tregs and subsequent activation of the protein phosphatase 2A (PP2A) complex (47). Sphingosine is derived from the enzymatic hydrolysis of sphingomyelin. Inside cells, sphingosine is phosphorylated by sphingosine kinase 1 (SPHK1) to form sphingosine-1-phosphate (S1P). As an important bioactive lipid mediator, S1P is widely involved in the regulation of various physiological processes, including inflammatory responses and lymphocyte migration (48).

During autophagy, the synthesis and processing of the autophagy marker LC3 are significantly enhanced, and its expression level is positively correlated with autophagic activity. Upregulation of LC3 expression reflects enhanced autophagic activity in Tregs. Under inflammatory conditions, autophagy enhances the survival of Tregs by removing damaged mitochondria, maintaining mitochondrial membrane potential, and reducing apoptotic signals. Simultaneously, autophagy stabilizes Foxp3 expression and prevents Tregs from converting toward a Th17 phenotype, ensuring the continuous exertion of their immunosuppressive functions. Therefore, autophagy enhancement mediated by LC3 upregulation is a key mechanism by which Tregs maintain homeostasis and function in the inflammatory microenvironment. This discovery provides a theoretical basis for targeting autophagy to regulate Treg function (49, 50). A study has shown that S1P can increase LC3 expression, correct autophagy dysfunction in Tregs, and restore their function and numbers. Additionally, S1P increases Ki67 expression in Tregs, promoting their proliferation, while downregulating IFN-γ expression and upregulating IL-10 and IL-13 expression, thereby inhibiting the inflammatory response. Consequently, S1P reduces lymphocyte infiltration in the submandibular glands of NOD mice, lowers anti-SSA/Ro and anti-SSB/La antibody levels, decreases organ indices, and increases salivary flow (51). Deltex1 was initially discovered in fruit flies and functions as an E3 ubiquitin ligase in the Notch signaling pathway. In mice, Deltex1 is a transcriptional target of nuclear factor of activated T cells (NFAT) and can promote T cell anergy (52). In patients with SjD, Deltex1 expression is significantly decreased. Further analysis revealed that Deltex1 expression levels in T cells are negatively correlated with the degree of fatigue, disease activity index (ESjDDAI), and patient-reported index (ESjDPRI). A study has shown that reducing Deltex1 expression can lower the proportion of CD4+ FoxP3+ Treg cells and inhibit Treg function and activity. Therefore, increasing Deltex1 expression may represent a potential therapeutic strategy for SjD by promoting Treg proliferation and function (53). In the experimental autoimmune encephalomyelitis (EAE) model, transient B-cell depletion or apoptosis of immune cells, combined with a single low-dose gamma-ray irradiation and administration of self-antigen peptides, successfully induced antigen-specific Tregs, thereby inhibiting disease progression (54). A subsequent study showed that anti-CD4 monoclonal antibodies combined with the self-antigen peptide Ro480 can promote the generation of SjD/Ro antigen-specific Tregs *in vivo*, inhibit IFN-γ production by CD4+ T cells, and alleviate inflammatory infiltration and tissue pathological damage. Additionally, this combined treatment upregulates the expression of aquaporin 5 (AQP5), inositol triphosphate receptor 3 (ITPR3), and sodium-potassium-chloride cotransporter 1 (SLC12A2), thereby improving salivary gland function and increasing saliva secretion in NOD mice (55). Mesenchymal stem cells (MSCs) have been extensively studied due to their excellent safety profile and therapeutic potential in various autoimmune diseases (56). They also demonstrate potential efficacy in treating SjD (57). MSCs exert immunomodulatory effects primarily by regulating CD4+ T cell subset proportions and inducing a local immunosuppressive microenvironment, and they also possess the capacity to differentiate into salivary gland epithelial cells (58). Umbilical cord-derived mesenchymal stem cells (UC-MSCs) offer advantages such as easy acquisition and low immunogenicity, making them a promising cell source for SjD treatment. Human UC-MSCs can upregulate Foxp3 and IL-10 expression, promote the differentiation of CD4+ Foxp3+ Tregs, and increase the proportion of Tregs. Concurrently, they downregulate the expression of pro-inflammatory cytokines such as TNF-α, IL-6, and IFN-γ, alleviate inflammatory infiltration and pathological damage in the submandibular and salivary glands of NOD mice, and significantly increase salivary secretion (59). Adipose-derived mesenchymal stromal cells (ADSCs) are a subtype of MSCs that can differentiate into osteoblasts and adipocytes while regulating immune responses, thereby alleviating inflammation or autoimmune diseases (60, 61). Interleukin-33 (IL-33) is a key factor that influences both innate immunity and adaptive immunity (62). A study has shown that IL-33 derived from ADSCs can increase the number of CD4+Foxp3+ Tregs, promote Treg proliferation, significantly alleviate submandibular gland inflammation, restore glandular function, and increase salivary flow in NOD mice (63). Interleukin-2 (IL-2) is a key cytokine that maintains immune homeostasis. Upon binding to its receptor, IL-2 initiates downstream signal transduction, leading to phosphorylation and activation of the transcription factor STAT5 (64). Dysfunction of the IL-2 signaling pathway and the resulting impairment of Treg function play crucial roles in the development of autoimmune diseases (65). Multiple studies have confirmed that IL-2 and its receptors play significant roles in the pathogenesis of SjD. Levels of soluble IL-2 receptor (sIL-2R) in the serum and saliva of SjD patients are significantly elevated and have been regarded as important indicators of disease activity (66). A cross-sectional study further revealed that elevated plasma sIL-2R levels in patients with SjD were significantly correlated with marked decreases in salivary flow. Following IL-2 stimulation, the proportion of pSTAT5+ cells within Tregs was significantly lower in patients compared with healthy controls, whereas no significant changes were observed in the frequencies of Tregs or pSTAT5 in conventional T cells. Additionally, the baseline proportion of pSTAT5+ Tregs was increased in patients and was significantly associated with positive serum autoantibodies and elevated plasma sIL-2R levels (67). These findings indicate that patients with SjD exhibit impaired IL-2/IL-2R signaling, resulting in reduced immunosuppressive capacity of Tregs. This impairment may promote lymphocyte infiltration into the salivary glands, thereby triggering clinical symptoms such as dry mouth.

### 4.2 Restoration of the Th17/Treg immune balance for SjD therapy

Studies have shown that human umbilical cord mesenchymal stem cells (UC-MSCs), UC-MSC-derived exosomes (UC-MSC-exo), exosomes derived from labial gland mesenchymal stem cells (LGMSC-exos), and dental pulp stem cells (DPSCs) can regulate the expression of Th17 and Treg-related factors, thereby restoring the Th17/Treg immune balance. These interventions synergistically improve intestinal microbiota composition, reduce inflammatory responses, inhibit lymphocyte infiltration, and ultimately exert anti-SjD effects. Mesenchymal stem cells (MSCs) have emerged as a promising therapeutic option for SjD owing to their low immunogenicity and potent immunomodulatory capabilities (68, 69). MSCs transplantation from various sources has demonstrated therapeutic efficacy in SjD models and clinical contexts (70). For instance, bone marrow-derived MSCs (BMMSCs) and dental pulp stem cells (DPSCs) have been shown to modulate immune cells, including T cells, B cells, natural killer cells, and dendritic cells; a property that has been validated in both experimental and clinical studies of autoimmune diseases (71). Notably, human umbilical cord-derived MSCs (UC-MSCs) and their exosomes (UC-MSC-exo) can upregulate Foxp3 expression, increase the proportion of Tregs, reduce IL-17 expression, decrease the proportion of Th17 cells, and restore the Th17/Treg immune balance. Moreover, they downregulate the expression of pro-inflammatory cytokines such as IFN-γ, TNF-α, IL-6, and IL-2, while upregulating IL-10 and TGF-β1, effectively alleviating inflammatory responses. In animal models, UC-MSCs and UC-MSC-exo increase the diversity and richness of the intestinal microbiota, improve intestinal homeostasis in NOD mice, reduce anti-Ro/SjDA antibody and α-Fodrin IgA levels, alleviate submandibular gland inflammatory infiltration, and ultimately significantly increase salivary flow rate (72). Labial gland-derived MSCs (LGMSCs) constitute a population of resident stem cells in the labial gland. The exosomes released by these cells contain various bioactive molecules and are considered an extension of MSCs (73). Specifically, LGMSC-derived exosomes (LGMSC-exos) can upregulate IL-10 expression, promoting Treg proliferation; downregulate IL-17 expression, inhibiting Th17 cell differentiation; restore the Th17/Treg immune balance; reduce the expression of IL-6 and IFN-γ, alleviating inflammatory responses; and ultimately reduce salivary gland inflammatory infiltration and pathological damage while increasing salivary flow in NOD mice (17). Similarly, dental pulp stem cells (DPSCs) can upregulate IL-10 and TGF-β1 expression, increase Treg numbers, downregulate IL-17a expression, reduce the proportion of Th17 cells, restore the Th17/Treg immune balance, lower IL-4 expression, alleviate submandibular gland inflammatory infiltration and pathological damage, and increase salivary flow (74). The conditioned medium from dental pulp stem cells (DPSC-CM) exhibits immunomodulatory properties, promotes tissue repair, and demonstrates anti-inflammatory effects comparable to those of bone marrow-derived mesenchymal stem cell conditioned medium (BMMSC-CM) (75). Specifically, DPSC-CM can upregulate IL-10 and TGF-β1 expression, increase Treg proportion, downregulate IL-17a expression, reduce Th17 cell proportion, restore the Th17/Treg immune balance, lower IL-4 and IL-6 expression, alleviate submandibular gland inflammatory responses, and increase salivary flow (76). The dynamic interplay between gut microbiota, intestinal epithelium, and mucosal immunity is essential for immune homeostasis, acting as a double-edged sword in inflammatory diseases by exerting both protective and pathogenic effects (77, 78). In SjD, inflammatory responses and reduced exocrine secretion may compromise gastrointestinal epithelial integrity and barrier function, disrupting host–microbiota immune crosstalk (79). The gut microbiota in SjD patients is skewed toward a pro-inflammatory state, with an expansion of pathobionts (e.g., *Escherichia coli*) at the expense of key anti-inflammatory and barrier-protective genera such as *Ruminococcus* and *Bifidobacterium*. This dysbiosis reduces FOXP3 expression, impairs Tregs differentiation, elevates IL-17 levels and Th17 proportion, upregulates pro-inflammatory cytokines including TNF-α, IL-6, IL-12, and IFN-γ, and downregulates IL-10, collectively exacerbating inflammatory responses in SjD (80). Therefore, promoting beneficial bacteria while inhibiting pathogenic ones can enhance Treg proliferation/activation and suppress Th17 activity, thus restoring the Th17/Treg immune balance. Ultimately, this approach may offer a novel therapeutic strategy for SjD. Ubiquitin-specific peptidase 18 (USP18) may be a key regulatory factor in SjD. USP18 is highly expressed in SjD patients and is associated with Th17/Treg imbalance. Upregulation of USP18 expression can increase Foxp3 expression and Treg proportion, reduce IL-17A and RORγt expression and Th17 proportion, restore the Th17/Treg immune balance, inhibit glandular lymphocyte infiltration, and increase salivary flow in NOD mice (81). Trichloroammonium (dioxirane-O,O’) thioammonium (AS101) is a non-toxic organic tellurium compound with broad immunomodulatory properties (82). It has demonstrated significant therapeutic efficacy in various systemic autoimmune diseases, including autoimmune encephalomyelitis, rheumatoid arthritis, and inflammatory bowel disease (83). A study has shown that AS101 can inhibit the expression of the transcription factor NFATc2, downregulate T-bet and RORC expression as well as IFN-γ and IL-17 production, and inhibit Th1/Th17 cell differentiation. Additionally, it upregulates Foxp3 expression, promotes Treg generation, restores the Th1/Th17/Treg immune balance, reduces TNF-α, IL-1β, and IL-6 expression, alleviates inflammatory responses, protects the corneal subbasal nerve, and improves dry eye symptoms (84). Fenofibrate, a synthetic peroxisome proliferator-activated receptor alpha (PPARα) agonist, is widely used in clinical practice for its potent lipid-lowering effects, reducing both cholesterol and triglyceride levels (85). It exhibits immunomodulatory activity in experimental autoimmune myocarditis models, attenuating inflammation by suppressing Th17 cell development and promoting Treg differentiation (86). However, its role and mechanisms in SjD remain unclear. Liver X receptor beta (LXRβ), as an important member of the nuclear receptor family, plays a crucial role in lipid metabolism, inflammation regulation, and immune homeostasis maintenance. Activation of LXRβ can affect T cell differentiation and function, and by regulating the Th17/Treg immune balance, it participates in the progression of autoimmune diseases. Therefore, it is regarded as a potential therapeutic target (87). Fenofibrate can increase the expression of PPAR-α and LXR-β, activate the PPAR-α/LXR-β signaling pathway, significantly upregulate Foxp3 expression, and increase Treg proportion. It also downregulates T-bet, IFN-γ, RORγt, and IL-17 expression, reduces Th1 and Th17 cell proportions, and restores the Th17/Treg immune balance, thereby reducing lymphocyte infiltration in the lacrimal glands, increasing tear secretion, and decreasing corneal fluorescein staining (88). Metformin exerts anti-inflammatory and immunomodulatory effects through activation of the AMPK signaling pathway (89). A study has shown that metformin can reduce the number of CD4+IL-17+pSTAT3(Y705)+ cells in the spleen, increase the number of CD4+Foxp3+CD25+ cells, decrease the Th1/Th17 ratio, increase the Treg ratio, and restore the Th1/Th17/Treg immune balance. It also inhibits the differentiation of CD4+CXCR5+Bcl6+IL-17+ cells, promotes the differentiation of CD4+CXCR5+Bcl-6+Foxp3+ cells, and restores the Tfh/Tfr ratio. Furthermore, it inhibits B cell differentiation into germinal center B cells, reduces total serum IgG, IgG1, and IgG2a levels, downregulates TNF-α, IL-6, and IL-17 expression, alleviates salivary gland inflammatory responses, improves gland function, and restores salivary flow (90). Nitrate is concentrated and reabsorbed by salivary glands, and its deficiency has been linked to salivary gland dysfunction. Supplementation with nitrate improves salivary gland activity in estrogen-deficient and radiation-induced injury models, suggesting its potential in preventing reduced saliva secretion (91, 92). Nitrate can regulate immune responses and maintain salivary gland function, but its preventive effect on SjD has not been fully explored. A study found that nitrate can inhibit the NF-κB pathway, promote Treg differentiation, inhibit Th17 cell differentiation, restore the Th17/Treg immune balance, reduce lymphocyte infiltration in salivary glands, restore gland function, and increase salivary flow in NOD mice (93). Interleukin-2 (IL-2) is a critical cytokine for the differentiation and maintenance of Foxp3+ Tregs (94). In NOD mice, the function of Tregs in the pancreas is impaired. However, IL-2 supplementation can restore their function and alleviate the severity of diabetes in these mice (95). Low-dose IL-2 (LDIL-2) can increase the number of Tregs and improve the symptoms of various autoimmune diseases (96, 97). In addition, LDIL-2 can increase the proportion of CD4+CD25+Foxp3+ Tregs, decrease the proportions of CD4+Bcl-6+PD-1+CXCR5+ Tfh cells, CD4+IFN-γ+ Th1 cells, and CD4+IL-17A+ Th17 cells, and restore the Th1/Th17/Tfh/Treg immune balance. It also lowers ANA, anti-SSA/Ro, and anti-SSB/La antibody levels, inhibits lymphocyte infiltration in the submandibular gland, and restores salivary flow (98). Homeodomain-interacting protein kinase 2 (HIPK2) is closely associated with dysregulated signaling pathways in Th17 cells, suggesting a key role in their development in SjD (99). Meanwhile, Runt-related transcription factor 1 (RUNX1), as a regulatory factor that can activate or inhibit transcription depending on the cellular microenvironment, is also an important regulatory molecule in Th17 cell differentiation (100). Diosgenin can upregulate RUNX1 expression, downregulate HIPK2 expression, increase Treg proportion, reduce Th17 cell proportion, and restore the Th17/Treg immune balance. It also lowers TNF-α, IL-6, and IL-17 expression, alleviates inflammatory responses, reduces lymphocyte infiltration, and restores salivary flow in NOD mice (101). The colonization factor antigen I (CFA/I) fibrous fimbriae of enterotoxin-producing Escherichia coli contain adhesion proteins that can prevent various autoimmune diseases in an antigen-independent manner (102). This effect is mainly achieved by inducing the production of regulatory cytokines such as IL-10, IL-13, IL-35 and TGF-β, thereby driving the differentiation and function of Tregs (103, 104). Lactic acid bacteria can easily express exogenous antigens through recombinant expression, which can activate the immune response, inhibit inflammatory reactions and slow down tumor proliferation, demonstrating a promising intervention potential (105). A study has shown that Lactococcus lactis expressing CFA/I fimbriae (LL-CFA/I) can significantly increase the expression of Foxp3, IL-10, and TGF-β, as well as the proportion of Tregs. Conversely, it reduces the expression of IL-17 and the proportion of Th17 cells, thereby restoring the Th17/Treg immune balance. Additionally, LL-CFA/I decreases the expression of pro-inflammatory cytokines such as IL-6 and IFN-γ, alleviates glandular inflammatory infiltration, and restores salivary flow rate in NOD mice (106). (The mechanisms of action of Treg-targeted therapies in SjD are summarized in **Table 1**).

**Table 1:** Therapeutic Strategies Targeting Tregs in SjD.

Regulation of Treg function	Intervention/agent	Model/population	Mechanism of Treg modulation	Therapeutic outcome in SjD	References
Regulation of Treg Proliferation and Activation	S1P	NOD mice	Treg function, number, and proliferation ↑	Inflammatory response, lymphocyte infiltration, anti-SSA/Ro and anti-SSB/La antibodies, and organ index ↓; salivary flow ↑	(51)
	Deltex1 expression ↓	SjD patients	CD4+Foxp3+ Treg proportion, function, and activity ↓	Fatigue score, ESSDAI, and ESSPRI ↑	(53)
	Anti-CD4 monoclonal antibody protein and Ro480	NOD mice	Treg proliferation ↑	Inflammatory infiltration and pathological damage ↓; salivary gland function and salivary flow ↑	(55)
	Human UC-MSC	NOD mice	Foxp3 and IL-10 expression ↑, CD4+Foxp3+ T cell differentiation ↑, Treg proportion ↑	Inflammatory infiltration and pathological damage ↓; salivary flow ↑	(59)
	IL-33 derived from ADSCs	NOD mice	CD4+Foxp3+ Treg proportion ↑, Treg proliferation ↑	Inflammatory response ↓; salivary gland function and salivary flow ↑	(63)
	IL-2/IL-2R ratio ↓	SjD patients	Treg immunosuppressive function ↓	Lymphocytic infiltration of salivary gland ↑; xerostomia ↑	(67)
Restoration of the Th17/Treg Immune Balance	Human UC-MSCs, UC-MSCs-exo	SjD patients; NOD mice	Foxp3 expression and Treg proportion ↑; IL-17 expression and Th17 proportion ↓	Inflammatory response ↓; salivary flow ↑	(72)
	LGMSC-Exos	NOD mice	IL-10 expression ↑, Treg proliferation ↑; IL-17 expression ↓, Th17 differentiation ↓	Inflammatory response and pathological damage ↓; salivary flow ↑	(17)
	DPSC	NOD mice	IL-10 and TGF-β1 expression ↑, Treg proportion ↑; IL-17a expression ↓, Th17 proportion ↓	Inflammatory response and pathological damage ↓; salivary flow ↑	(74)
	DPSC-CM	NOD mice	IL-10 and TGF-β1 expression ↑, Treg proportion ↑; IL-17a expression ↓, Th17 proportion ↓	Inflammatory response ↓; salivary flow ↑	(76)
	*Beneficial bacteria* ↓; *Escherichia coli* ↑	SjD patients	Foxp3 expression ↓, Treg proportion ↓; IL-17 expression ↑, Th17 proportion ↑	Inflammatory response↑	(80)
	USP18 expression ↑	NOD mice	Foxp3 expression ↑, Treg proportion ↑; IL-17A and RORγt expression ↓, Th17 proportion ↓	Lymphocytic infiltration of salivary gland ↓; salivary flow ↑	(81)
	AS101	NOD mice	Foxp3 expression ↑, Treg proportion ↑; T-bet, RORγt, IFN-γ, and IL-17 expression ↓, Th1/Th17 proportion ↓	Inflammatory response ↓, corneal nerve protection ↑, dry eye symptoms ↓	(84)
	Fenofibrate	SjD patients	Foxp3 expression ↑, Treg proportion ↑; T-bet, IFN-γ, RORγt, and IL-17 expression ↓, Th17 proportion ↓	Lymphocyte infiltration and inflammatory response ↓; tear secretion ↑	(88)
	Metformin	SjD patients	Treg proportion ↑, Th1/Th17 proportion ↓	Inflammatory response ↓; salivary gland function and salivary flow ↑	(90)
	Nitrate	NOD mice	Treg differentiation ↑, Th17 differentiation ↓	Lymphocyte infiltration ↓; salivary gland function and salivary flow ↑	(93)
	LDIL-2	NOD mice	Treg proportion ↑; Th17 proportion ↓	Inflammatory response ↓; salivary flow ↑	(98)
	Diosgenin	NOD mice	Treg proportion↑, Th17 proportion↓	Inflammatory response and lymphocytic infiltration ↓; salivary flow ↑	(101)
	LL-CFA/I	NOD mice	Foxp3, IL-10, and TGF-β expression ↑, Treg proportion ↑; IL-17 expression ↓, Th17 proportion ↓	Inflammatory infiltration ↓; salivary flow ↑	(106)

↑, Increase/Activate/Enhance; ↓, Decrease/Lower/Inhibit)(The strategies listed in this table are all at the preclinical or early clinical exploration stage. The applicable population is inferred based on mechanism studies, and clinical application requires further stratification; safety and risk assessment only summarizes the current main concerns, and have not been verified by large-scale clinical trials. This table is intended to present the research direction, rather than providing guidance for clinical decision-making.

## 5 Discussion

In SjD, dysfunction of Tregs manifested as both numerical deficiency and functional impairment disrupts immune tolerance, thereby serving as a key driver of inflammation and autoantibody production. Therefore, restoring Treg homeostasis is regarded as an important therapeutic strategy. Through analysis of the relevant literature, we draw the following conclusions: (1) Regarding regulatory mechanisms, most studies focus on restoring the Th17/Treg immune balance, while some focus on directly promoting Treg proliferation and activation to enhance their function. However, the proportion of Th17 cells remains underexplored in a subset of studies, creating a critical knowledge gap. This gap is particularly notable given that restoring the Th17/Treg immune balance is a major focus of current SjD research. (2) Some studies explore negative regulatory mechanisms affecting Tregs, such as downregulating Deltex1 expression, altering intestinal microbiota composition (reducing *beneficial bacteria* and increasing *Escherichia coli* abundance), and impairing IL-2/IL-2R signaling. These factors inhibit Treg proliferation and activation, thereby contributing to SjD pathogenesis. Therefore, reversing these negative regulatory effects on Tregs represents a promising therapeutic strategy for SjD. (3) Several commonly used clinical drugs show regulatory potential for Tregs, such as fenofibrate and metformin. In addition, the traditional Chinese medicine ingredient diosgenin also demonstrates therapeutic value in restoring the Th17/Treg immune balance. These findings underscore the therapeutic potential of Western medicine in SjD and highlight the growing significance of traditional Chinese medicine (TCM), offering a promising direction for future research. (4) Mesenchymal stem cells (MSCs) are important regulators of Treg function in SjD, including human UC-MSCs, LGMSC-Exos, ADSCs, and DPSCs. Among these, ADSCs increase the proportion of Tregs; human UC-MSCs both increase Treg proportion and restore the Th17/Treg immune balance; whereas LGMSCs and DPSCs primarily regulate the Th17/Treg immune balance. The comprehensive application of diverse MSCs thus represents a highly promising strategy for SjD, establishing MSC-based therapy as a pivotal direction for future drug development. (5) Clinical studies in SjD patients remain relatively limited, and most mechanistic studies are based on the NOD mouse model. Of these, human UC-MSCs and UC-MSC-derived exosomes have been shown to exert effects in both SjD patients and NOD mice. Notably, most studies cited in this article employed the classic NOD mouse model—the standard spontaneous model of SjD—to validate molecules that restore the Th17/Treg immune balance and increase Treg numbers. This model recapitulates key pathological features of human SjD, including salivary gland lymphocytic infiltration, autoantibody production, and secretory dysfunction, and its immunopathological processes are closely linked to Th17/Treg immune imbalance. Accordingly, it is widely used for mechanistic studies and efficacy evaluations of such molecules. However, reliance on a single model may limit the generalizability of findings. Furthermore, the preclinical models have inherent limitations in simulating the heterogeneity of human diseases, the immune background, and the disease progression stages. The comparability between their research endpoints and clinical endpoints also needs to be clarified. At the same time, issues such as the dose-effect relationship, the stability and safety characteristics of cell products, as well as the potential risk of systemic immunosuppression, still require systematic evaluation in larger-scale preclinical studies and gradually advancing early clinical trials. Given the heterogeneity of SjD, different animal models vary in pathogenesis, immune phenotype, and treatment response. Induced models (e.g., submandibular gland protein immunization) capture acute inflammatory processes, while genetically modified models (e.g., IL-14α transgenic mice) help delineate specific molecular pathways in disease pathogenesis. In conclusion, Tregs therapy is still in the early stage of transitioning from preclinical exploration to clinical application. The existing evidence provides a preliminary foundation for its subsequent research, but there is still a considerable distance from achieving a clear clinical value. Thus, future research should integrate multiple complementary models to systematically validate candidate molecules, thereby overcoming single-model limitations and enabling a more comprehensive assessment of their universality and clinical potential in treating SjD by restoring Th17/Treg immune balance. (6) In terms of clinical application prospects and the reproducibility of therapeutic effects, we believe that developing new drugs targeting IL-2 has significant advantages and is one of the most promising candidate strategies. Impairment of IL-2/IL-2R signaling can weaken the immunosuppressive function of Tregs, while supplementation with low-dose IL-2 can restore Th17/Treg immune balance. This therapeutic effect has been verified in patients with SjD. Moreover, clinical studies have shown that low-dose IL-2 treatment can significantly increase the proportion of Tregs, improve the SjD disease activity score, and has a good safety profile. Mechanistically, low-dose IL-2 selectively activates Tregs by preferentially binding to high-affinity IL-2 receptors and inhibits excessive activation of effector T cells. These findings highlight the important value of targeting the IL-2 signaling pathway in the treatment of SjD and provide a strong theoretical basis for the development of new drugs targeting IL-2.

Regarding current and future Treg-targeted treatment strategies for SjD, several drugs already used in clinical practice may exert their effects, at least in part, through Treg modulation. For instance, hydroxychloroquine has been reported to increase Treg proportion and enhance suppressive function; glucocorticoids can induce Treg expansion, though high doses may compromise their stability; and rituximab treatment leads to a relative increase in Treg proportion, which may correlate with clinical response. These findings indicate that existing therapies already involve Treg regulation, yet direct Treg-targeted interventions remain lacking. Such strategies have shown unique therapeutic promise. Low-dose IL-2, for instance, selectively expands Tregs to restore immune balance in various autoimmune diseases and has demonstrated potential to improve disease activity in early-phase studies of SjD. Treg adoptive transfer, although still preclinical, offers a cellular treatment option for severe or refractory patients. Unlike broad-spectrum immunosuppression, these approaches aim to rebuild immune homeostasis rather than simply suppress immune responses, potentially yielding more durable efficacy with fewer infection-related adverse effects.

However, current research on Treg-based therapies for SjD has several notable limitations: (1) Mechanistic insights remain superficial. Most studies have focused on describing the Th17/Treg imbalance phenotype, with insufficient attention to upstream transcriptional regulation, metabolic reprogramming, epigenetic mechanisms, and the interaction network between Tregs and other immune cells. (2) Translational evidence is constrained by reliance on animal models. Clinical studies in patients are scarce, limiting the evaluation of therapeutic strategies. (3) Intervention strategies are largely confined to promoting Treg proliferation or exogenous Treg infusion, with limited exploration of multi-target or integrated approaches. (4) Treg heterogeneity and functional plasticity remain underexplored, and the roles of distinct Treg subpopulations in disease pathogenesis require further clarification. (5) Traditional Chinese medicine and natural products remain underexploited in this field, despite their potential multi-component, multi-pathway advantages. (6) Biomarker development is lacking. Specific biomarkers that dynamically reflect Treg functional recovery and immune reconstitution are absent, and current efficacy evaluation systems are inadequate for precise individualized treatment. Collectively, these limitations impede the advancement and clinical translation of Treg-targeted therapeutic strategies for SjD.

Future research should focus on establishing a multi-level, systematic research framework. At the mechanism level, multi-omics technologies should be employed to analyze the upstream regulatory network of Treg dysfunction (including transcriptional, metabolic, and epigenetic mechanisms) in depth and to clarify its dynamic interaction network with other immune cells, such as B cells and macrophages. In terms of clinical translation, large-scale patient cohorts should be established to promote rigorous clinical trials of low-dose IL-2, mesenchymal stem cells, and other Treg-targeted strategies, accelerating the transition from discovery in animal models to clinical applications. In terms of treatment strategies, efforts should move beyond the single intervention model to develop multi-target integrated therapies that combine immune regulation, microbiota intervention, and metabolic regulation, while emphasizing the precise modulation of distinct Treg functional subsets. At the same time, research into the multi-component, multi-pathway regulatory effects of traditional Chinese medicine should be strengthened to explore its potential to modulate immune balance through the Treg network. Additionally, it is urgent to develop specific biomarkers that can dynamically reflect functional Treg reconstitution, to construct a multi-dimensional efficacy evaluation system that includes immune microenvironment indicators, and to actively introduce new technologies such as organoid models and artificial intelligence analysis. Through these systematic efforts, it is expected that the treatment of SjD will achieve a fundamental transformation from symptom control to immune reconstitution.
